# The phenomenology and treatment of idiopathic adult-onset truncal dystonia: a retrospective review

**DOI:** 10.1186/s40734-016-0044-9

**Published:** 2016-10-24

**Authors:** Debra J. Ehrlich, Steven J. Frucht

**Affiliations:** Icahn School of Medicine at Mount Sinai, 5 East 98th Street, 1st Floor, Box 1637, New York, NY 10029 USA

**Keywords:** Truncal, Axial, Dystonia, Adult

## Abstract

**Background:**

Focal dystonia is the most common type of adult-onset dystonia; however, it infrequently affects truncal musculature. Although commonly attributed to secondary etiologies such as a neurodegenerative illness or tardive syndromes, the entity of idiopathic adult-onset truncal dystonia has only been previously described in a few case reports and small case series. Here we characterize seven cases of adult-onset primary truncal dystonia and present them within the scope of the existing literature.

**Methods:**

Retrospective chart review of medical records and patient videos of seven adult patients with idiopathic truncal dystonia evaluated by the senior movement disorder neurologists in an urban outpatient clinic.

**Results:**

The mean age of onset of idiopathic truncal dystonia was 47.6 years old and the majority of patients were male. Truncal flexion was the most common direction of dystonic movement and the dystonia was most frequently induced by action and could be improved by use of a sensory trick. The majority of patients were refractory to 3 or more oral treatments and only two patients exhibited significant functional improvement with botulinum toxin injections. One patient enjoyed significant benefit with bilateral internal globus pallidus deep brain stimulation.

**Conclusions:**

Although a relatively rare presentation, patients with idiopathic adult-onset truncal dystonia can be identified by a common phenomenology. Diagnosis of this highly disabling condition is important because these patients are frequently refractory to multiple oral treatments and may benefit from early treatment with botulinum toxin or deep brain stimulation.

**Electronic supplementary material:**

The online version of this article (doi:10.1186/s40734-016-0044-9) contains supplementary material, which is available to authorized users.

## Background

Focal dystonia is the most common type of adult-onset dystonia, and dystonia may affect the face, voice, hand or leg. Aside from the neck, dystonia rarely affects axial musculature unless associated with exposure to a dopamine receptor blocker or as a feature of a neurodegenerative illness. Truncal dystonia is characterized by involuntary contractions and postures of the paraspinal, abdominal or chest muscles. Idiopathic truncal dystonia of adult onset has only been previously described in a few case reports and small case series.

Here we report seven cases of adult-onset dystonia involving primarily the axial musculature. We also compare the current case series to previous reports of idiopathic adult-onset truncal dystonia and review the challenges of designing effective treatments for this condition.

## Methods

We conducted a retrospective review of medical records and patient videos of patients with the diagnosis of idiopathic truncal dystonia, evaluated by senior movement disorder physicians in the outpatient clinic of an urban academic center between 1/2009 and 1/2016. Patients were included if symptom onset occurred over 18 years of age and they exhibited primary dystonia of the trunk or axial muscles. Cases of secondary dystonia or tardive syndromes were excluded. The study was approved by the Institutional Review Board of the Mount Sinai School of Medicine. Written informed consent for videotaping and use of videos in research and publication was obtained prior to video recording.

## Results

Seven patients with a primary diagnosis of idiopathic truncal dystonia were evaluated in the movement disorders clinic between 1/2009 and 1/2016. Within this group of patients, the mean age of onset of truncal dystonia was 47.6 years (SD 9.2). Six patients were male (85.7 %). Patients were symptomatic for an average of 4.7 years (SD 4.8) before their initial visit. Most patients presented with a chief complaint of pulling or tightness of the muscles in the back, abdomen or trunk. There was no history of neuroleptic exposure and no known family history of dystonia in any of the patients. All patients had a spinal MRI (see Table 1) and two patients (28.6 %) had a history of spinal surgery, though symptom onset preceded the operative procedure in one patient (Table 1, case 6). There were no myopathic findings in any of the patients. Additionally, examination of the abdomen did not reveal evidence of involuntary movements consistent with belly dancer dyskinesia. Anterior flexion of the trunk was the predominant direction of dystonic movement in four patients (57.1 %) while lateral flexion was seen in one patient (14.3 %) and truncal extension was the primary direction of involuntary movement in two patients (28.6 %). In most patients the dystonic movements were isolated to the musculature of the trunk; however, associated movements of a shoulder were seen in 2 patients. In more than half of patients (57.2 %), dystonia was present only with specific voluntary movements or action, while dystonia was present at rest though worsened by action in the remainder. In most patients, standing or walking provoked dystonic movements. Additionally, most patients (71.4 %) were aware of a specific sensory trick that suppressed the dystonic movement. Common sensory tricks included running, placing hands in the pants pockets, or tucking hands in the posterior pants waistband. Video 1 (Additional file 1) includes short samples demonstrating the characteristic dystonic truncal movements seen in each patient in this case series.

**Table 1:** Summary of demographics and clinical features in case series

Case #	Sex	Age of onset	Presenting complaint	Family history of dystonia/genetic testing	Spine imaging or history of trauma	Exposure to dopamine depleting/blocking agents	Primary axial movement	Secondary axial movement	Involvement of other body regions	Action vs rest	Provoking positions or actions	Sensory trick	Treatment response
1	M	64	Pulling sensation of lower abdominal muscles	No/negative 14 gene dystonia-dyskinesia panel	MRI T/L spine: left L4/L5 herniated disc, exaggerated kyphosis of thoracic spine	No	Flexion	Slight right lateral tilt	No	Action	Standing, walking	Running, dancing, hands in posterior waistband of pants	BAC-small improvement THP (max dose unknown), CARB/LEVO, BTX-no benefit
2	M	44	Tightness and pulling in left lower back	No/no	MRI C/T/L spine: mild disc herniation and osteophytic changes, no cord pathology	No	Left lateral flexion	None	Downward left shoulder movement	Action	Walking or turning	Running	THP (6 mg/day), BTX-small improvement
3	M	43	Abdominal contractions	No/no	L4/L5 fusion for degenerative disc disease, 6 months post-op developed involuntary abdominal contractions	No	Flexion	None	Anterior right shoulder movement	Rest, worse with action	Sitting, worsened by standing or walking	None	BTX-50 % benefit CNZ, CARB/LEVO-no benefit THP (9 mg/day)-small benefit OXC, GBP, PGB-transient benefit
4	M	54	Muscle spasms in chest	No/no	MRI C/T/L spine: mild DJD, no cord pathology	No	Extension	None	No	Rest, worse with action	Supine, reclining, walking	None	BAC, THP (4 mg/day), CNZ, BTX-no benefit
5	M	47	Abnormal pelvic movements and gluteal clenching while standing	No/no	MRI C/T/L spine: no pathology	No	Flexion	Left lateral tilt	No	Action	Standing	Marching, running	CNZ-modest benefit THP (2 mg/day)-no benefit BAC, BTX-small benefit
6	M	46	Forward flexion of trunk when walking	No/no	MRI L spine: L4/L5 stenosis and mild-moderate disc herniation/ after symptom onset had L3-L5 laminectomy and L4/L5 disc micro-dissection with improvement in pain but no change in dystonia	No	Flexion	None	No	Action	Walking, running, going up/down stairs	Hands in pockets, holds hands against torso with mild pressure	BTX-70 % improvement THP (10 mg/day), BAC-no benefit
7	F	35	Pulling of the trunk backwards and to the right	No/no	MRI L spine: DJD at L4/L5 and L5/S1, no cord pathology	No	Extension	Right lateral tilt	No	Rest, worse with action	Writing, worsened by standing or walking	Running, leaning against wall, lying on stomach, voluntary inversion of right leg while walking	DBS-excellent benefit THP (12 mg/day), BAC, CARB/LEVO, LEV, BTX, HYZ-no benefit

*Abbreviations*: *C* cervical, *T* thoracic, *L* lumbar, *DJD* degenerative disc disease, *FHx* family history, *BAC* baclofen, *THP* trihexyphenidyl (the maximal daily dose of THP tried in each patient is noted in parentheses), *BTX* botulinum toxin, *CNZ* clonazepam, *CARB*/*LEVO* carbidopa/levodopa, *OXC* oxcarbazepine, *GBP* gabapentin, *PGB* pregabalin, *LEV* levetiracetam, *HYZ* hydroxyzine, *DBS* deep brain stimulation


Compilation video of patients with truncal dystonia. Brief representative samples demonstrating the characteristic dystonic truncal movements exhibited by each patient in this case series are shown. The first patient exhibits primarily truncal flexion with subtle lateral tilt to the right; the second clip demonstrates left lateral flexion and the third patient experiences truncal flexion exacerbated by walking. The fourth clip shows truncal extension in contrast to the fifth patient who exhibits truncal flexion with mild left lateral tilt. The sixth clip demonstrates truncal flexion while the last case expresses primarily truncal extension with right lateral tilt. Of note, per the patient’s report, the abnormal postures of the right hand and leg seen in case 7 are voluntary mechanisms she employed to compensate for her axial dystonia.


Most patients proved refractory to multiple oral medications. In fact, five patients (71.4 %) were tried on three or more oral treatments (see Table 1) with minimal to no benefit. All seven patients were treated with trihexyphenidyl; however only two patients exhibited mild improvement in their dystonia while the other five had no benefit. Poor tolerability to low dose trihexyphenidyl limited dose escalation in three of the patients who showed no benefit (see Table 1 for maximum dose of trihexyphenidyl used in each patient). Similar findings were observed in the five patients treated with baclofen, with a mild benefit seen in 40 % and no benefit in the other 60 %. Botulinum toxin injections were attempted in all seven patients, with two patients (28.5 %) who exhibited at least 50 % functional improvement after injections. In both patients who exhibited significant functional improvement after injections, each received a total of 700U of onabotuliumtoxinA divided between multiple sites in the bilateral rectus abdominis muscles with continuing benefit for at least 1 year. All patients who showed functional benefit after botulinum toxin injections exhibited flexion as the primary direction of axial movement while no improvement was seen in the two patients with the extensor phenotype. One patient (Table 1, case 5) had deep brain stimulation (DBS) to the bilateral internal globus pallidus (GPi) with significant functional improvement sustained for at least 3 months (at the time of writing this paper).

## Discussion

We summarize the clinical features and phenomenology of previously published case reports and case series in Table 2. The largest such series was reported by Bhatia et al, who described a group of 18 patients with primarily axial dystonia with adult or late adolescent onset. Our results are similar to Bhatia’s in that dystonia most commonly affected men in the fifth decade of life and flexion was the most common direction of involuntary truncal movements [1]. Other reports of idiopathic truncal dystonia are much smaller, although all prior published reports support an onset in the forth to sixth decades of life [1–5]. Also consistent with our findings, truncal flexion was the most common direction of dystonic movement while truncal extension and lateral bending were relatively rare [1–5]. When occurrence at rest compared to action was considered, our results are consistent with prior reports in that dystonia was precipitated or worsened by action [1, 2]. Also consistent with our findings, most patients employed a sensory trick to ameliorate their dystonia [1, 2].

**Table 2:** Comparison of prior published reports of idiopathic truncal dystonia

Case series/report	Year	No. of cases	Mean age of onset	Male %	Predominant truncal movement-flexion (%)	Predominant truncal movement-extension (%)	Predominant truncal movement-lateral (%)	Precipitated or worsened by action	Sensory trick
Bhatia et al. [1]	1997	18	41	55.6	55.6	22.2	5.6	In the majority	In some
Zittel et al. [2]	2009	1	36	0	100	0	100	Yes	-----
Sobstyl et al. [3]	2012	1	43	100	100	0	0	-----	-----
Shaikh et al. [4]	2014	4	55.5	50	50	50	0	-----	-----
Voos et al. [5]	2014	1	33	0	0	100	0	-----	-----
Current case series	2016	7	46.7	85.7	57.1	28.6	14.3	100 %	71.4 %

Similar to our case series, most prior reports found that patients with adult onset truncal dystonia were refractory to multiple oral medications [1–5]. While two patients in our case series benefited from botulinum toxin injections, few prior studies report response to botulinum toxin in idiopathic truncal dystonia. One patient with flexion dystonia of the trunk did enjoy transient relief of the dystonia of his upper thoracic region with type A botulinum toxin injections, however he ultimately developed resistance and injections were no longer effective [3]. Although pain relief was reported in some patients, other publications report no benefit with botulinum toxin [1, 2, 5]. Interestingly, in our case series, only patients with truncal flexion showed improvement after botulinum toxin injections while no benefit was seen in patients with truncal extension.

Bilateral GPi DBS has been previously shown to improve axial symptoms in patients with primary generalized dystonia [6] in addition to patients with truncal dystonia occurring as a result of secondary etiologies [7–10]. However, there are only a few case reports of the use of DBS in the treatment of primary truncal dystonia. Shaikh et al report a series of 4 patients with adult-onset axial dystonia who were treated with bilateral GPi DBS. All patients exhibited a substantial functional improvement in truncal dystonia with percent improvement in the Burke-Fahn-Marsden dystonia rating scale (BFMDRS) ranging from 72.4 to 100 % [4]. Another case of predominately adult-onset axial dystonia, although also with later spread to the neck and shoulders, demonstrated complete improvement in axial symptoms after 12 months of treatment with bilateral GPi stimulation. A similar patient with adult onset truncal dystonia exhibited a substantial improvement in axial dystonia with unilateral GPi stimulation (BFMDRS motor score decreased from 33 to 6 after 6 months) [3]. Our patient who was treated with bilateral GPi DBS stimulation showed a similar excellent response to GPi stimulation after 3 months.

Limitations of our case series include the fact that genetic testing was not completed on most patients. Additionally, while all patients denied prior exposure to neuroleptics, it is possible that a tardive case may have been inadvertently included. Given that this was a retrospective chart review, the treatments tried in each patient were not standardized, complicating direct comparisons. Similarly, botulinum toxin injections were not standardized, and different injectors employed different techniques, injected different muscle groups, and used different doses. We also do not have long-term data available on some patients, including the long-term response to DBS. Nevertheless, we believe that our patients add to the growing recognition of this syndrome.

## Conclusions

Identification of this highly disabling condition is important, as most patients are refractory to the typical oral treatments for dystonia. Early treatment with botulinum toxin or DBS might be considered to reduce the degree of functional impairment..

## Abbreviations

BFMDRS: Burke-Fahn-Marsden dystonia rating scale
DBS: Deep brain stimulation
GPi: Internal globus pallidus
